# An arterial spin labeling−based radiomics signature and machine learning for the prediction and detection of various stages of kidney damage due to diabetes

**DOI:** 10.3389/fendo.2024.1333881

**Published:** 2024-11-18

**Authors:** Feier Ma, Xian Shao, Yuling Zhang, Jinlao Li, Qiuhong Li, Haizhen Sun, Tongdan Wang, Hongyan Liu, Feiyu Zhao, Lianqin Chen, Jiamian Chen, Saijun Zhou, Qian Ji, Pei Yu

**Affiliations:** ^1^ National Health Commission (NHC) Key Laboratory of Hormones and Development, Chu Hsien-I Memorial Hospital and Tianjin Institute of Endocrinology, Tianjin Medical University, Tianjin, China; ^2^ Tianjin Key Laboratory of Metabolic Diseases, Tianjin Medical University, Tianjin, China; ^3^ Department of Radiology, Tianjin First Central Hospital, Tianjin, China; ^4^ Ultrasound Diagnostic Center, The First Hospital of Jilin University, Jilin, China; ^5^ Respiratory and Critical Care Medicine Department, The Third Medical Center of the People's Liberation Army General Hospital, Beijing, China

**Keywords:** radiomics signature, arterial spin labeling, texture analysis, diabetic kidney damage, machine learning (ML)

## Abstract

**Objective:**

The aim of this study was to assess the predictive capabilities of a radiomics signature obtained from arterial spin labeling (ASL) imaging in forecasting and detecting stages of kidney damage in patients with diabetes mellitus (DM), as well as to analyze the correlation between texture feature parameters and biological clinical indicators. Additionally, this study seeks to identify the imaging risk factors associated with early renal injury in diabetic patients, with the ultimate goal of offering novel insights for predicting and diagnosing early renal injury and its progression in patients with DM.

**Materials and methods:**

In total, 42 healthy volunteers (Group A); 68 individuals with diabetes (Group B) who exhibited microalbuminuria, defined by a urinary albumin-to-creatinine ratio (ACR)< 30 mg/g and an estimated glomerular filtration rate (eGFR) within the range of 60–120 mL/min/1.73m²; and 53 patients with diabetic nephropathy (Group C) were included in the study. ASL using magnetic resonance imaging (MRI) at 3.0T was conducted. The radiologist manually delineated regions of interest (ROIs) on the ASL maps of both the right and left kidney cortex. Texture features from the ROIs were extracted utilizing MaZda software. Feature selection was performed utilizing a range of methods, such as the Fisher coefficient, mutual information (MI), probability of classification error, and average correlation coefficient (POE + ACC). A radiomics model was developed to detect early diabetic renal injury, extract imaging risk factors associated with early diabetic renal injury, and examine the relationship between significant texture feature parameters and biological clinical indicators. Patients with DM and kidney injury were followed prospectively. The study utilized seven machine learning algorithms to develop a detective radiomics model and a comprehensive predictive model for assessing the progression of kidney damage in patients with DM. The diagnostic efficacy of the models in detecting variations in diabetic kidney damage over time was evaluated using the area under the curve (AUC) of the receiver operating characteristic (ROC) curve. Empower (R) was used to establish a correlation between clinical biological indicators and texture feature metrics. Statistical analysis was conducted using R, Python, MedCalc 15.8, and GraphPad Prism 8.

**Results:**

A total of 367 texture features were extracted from the ROIs in the kidneys and refined based on selection criteria using MaZda software across groups A, B, and C. The renal blood flow (RBF) values of the renal cortex in groups A, B, and C exhibited a decreasing trend, with values of 256.458 ± 54.256 mL/100g/min, 213.846 ± 52.109 mL/100g/min, and 170.204 ± 34.992 mL/100g/min, respectively. There was a positive correlation between kidney RBF and eGFR (r = 0.439, P<0.001). The negative correlation between RBF and various clinical parameters including urinary albumin-to-creatinine ratio (UACR), body mass index (BMI), diastolic blood pressure (DBP), blood urea nitrogen (BUN), and serum creatinine (SCr) was investigated. Through the use of a least absolute shrinkage and selection operator (LASSO) regression model, the study identified the eight most significant texture features and biological indicators, namely GeoY, GeoRf, GeoRff, GeoRh, GeoW8, GeoW12, S (0, 4) Entropy, and S (5, -5) Entropy. Spearman correlation analysis revealed associations between imaging markers in early diabetic patients with kidney damage and factors such as age, systolic blood pressure (SBP), Alanine Transaminase (ALT), Aspartate Amino Transferase (AST) albumin, uric acid (UA), microalbuminuria (UMA), UACR, 24h urinary protein, fasting blood glucose (FBG), two hours postprandial blood glucose (P2BG), and HbA1c. The study utilized ASL imaging as a detection model to identify renal injury in patients with DM across different stages, achieving a sensitivity of 85.1%, specificity of 65.5%, and an AUC of 0.865. Additionally, a comprehensive prediction model combining imaging labels and biological indicators, with the naive Bayes machine learning algorithm as the best model, demonstrated an AUC of 0.734, accuracy of 0.74, and precision of 0.43.

**Conclusion:**

ASL imaging sequences demonstrated the ability to accurately detect alterations in kidney function and blood flow in patients with DM. Strong associations were observed between renal blood flow values in ASL imaging and established clinical biomarkers. These values show promise in detecting early microstructural changes in the kidneys of diabetic patients. Utilizing image markers in conjunction with clinical indicators was effective in identifying early renal dysfunction and its progression in individuals with DM. Furthermore, the integration of imaging texture feature parameters with clinical biomarkers holds significant potential for predicting early renal damage and its progression in patients with diabetes.

## Introduction

Diabetic kidney disease (DKD) is a prevalent condition that increases the risk of cardiovascular disease and can lead to renal failure (1).

In individuals with chronic kidney disease (CKD), albuminuria and estimated glomerular filtration rate (eGFR) serve as predictive biomarkers for the progression of kidney dysfunction. However, previous studies suggest that the staging of proteinuria may not accurately predict renal function (2) in diabetic patients with normoalbuminuria who demonstrate preserved renal function and no apparent renal damage at different stages. Therefore, early detection of renal injury through sensitive imaging techniques is crucial.

Renal blood flow (RBF) can be measured non-invasively with arterial spin labeling magnetic resonance imaging (ASL MRI), a promising technique that holds considerable value in research (3–5). In ASL, an endogenous tracer is magnetically tagged and arterial blood water perfusion is measured by subtracting the labeled image from the non-labeled (control) image (6). RBF, tissue perfusion, oxidation, microstructure, inflammation, and fibrosis are crucial elements in the pathogenesis of various renal disorders, with biomarkers such as RBF being responsive to these alterations (4). Furthermore, quantitative assessment of renal blood perfusion by renal ASL-MRI can evaluate the protective effect of hypoglycemic drugs on kidney function (7). While prior research has concentrated on cortical perfusion ASL measurements for the detection of hemodynamic variations in early diabetic nephropathy, we argue that the analysis of texture features warrants further investigation.

Radiomics presents a non-invasive method for evaluating kidney damage in diabetic patients at various stages through the extraction of statistical information from multiple quantitative and analyzable medical images. Utilizing data-characterization algorithms, the extraction of numerous features from medical images can reveal digital disease fingerprints (8).

Through the use of labeled training samples, machine learning (ML) algorithms develop mathematical models to classify new data and generate predictions from unlabeled data (9). Radiomics bridges imaging (‘macroscopic’) and histology (‘microscopic’) and provides a new dimension for assessing cancer structures (10). In one study, texture-feature Gray Level Co-occurrence Matrix (GLCM) inverse difference was found to be the most representative feature (an avatar feature) in a model predictive of poor outcomes in patients treated with immunotherapy (11). Another study used cross-validation and grid search to assess three ML models [(XGBoost, Random Forest, and least absolute shrinkage and selection operator (LASSO)] for their ability to accurately predict the risk of malignancy for pulmonary nodules. They found the LASSO model yielded the best predictive performance in cross-validation (12). At present, there are no investigations on renal function in individuals with diabetes based on ASL imaging. In this study, patients with diverse levels of renal impairment were utilized to develop a precise and effective model for assessing radiomics texture characteristics through the application of machine learning methodologies.

Furthermore, this research expanded the examination to incorporate supplementary radiomics texture attributes in ASL imaging to ascertain whether imaging can detect renal injury earlier than conventional clinical markers. Additionally, the study aimed to predict the progression of renal impairment in diabetic individuals with normal clinical indicators. Advanced machine learning techniques were employed to uncover latent features within the radiomics data and explore the potential correlation between texture features and clinical biological indicators. It provides a rich opportunity for further research on the development of imaging omics in diabetic nephropathy.

## Materials and methods

### Study population

The study was approved by the ethics committee at Tianjin Medical University, with all participants providing informed consent prior to their involvement. Retrospective and prospective analyses were conducted on clinical and imaging data of individuals, including healthy volunteers, individuals with diabetes, and patients with diabetes, at Chu Hsien-I Memorial Hospital, Tianjin Medical University, from 1 January 2018 to 31 December 2022.

The diagnosis of diabetes mellitus (DM) adhered to the criteria outlined by the American Diabetes Association (13). Using the creatinine equation developed from the CKD-Epidemiology Collaboration (EPI) equation, the eGFR for each participant was calculated (14).

Group A, consisting of 42 healthy volunteers (24 men and 18 women, aged 28-72 years with a mean age of 46 years), exhibited normal eGFR levels and no indications of cardiac disease, gout, hypertension, or kidney disease.

Group B consisted of 68 patients with DM (39 men and 29 women, with an age range of 17-70 years and a mean age of 50 years). These patients demonstrated an eGFR between 60 and 120 mL/(min·1.73 m²) and an albumin-to-creatinine ratio (ACR)< 30 mg/g.

Group C included 53 diabetic patients (35 men and 18 women, with an age range of 33-74 years and a mean age of 55 years) who had an eGFR< 60 mL/(min·1.73m²) or an ACR>30 mg/g. The participants in groups B and C were clinically diagnosed with DM.

The study’s exclusion criteria encompassed patients with a history of kidney tumor, trauma, or surgery (two cases), those with renal morphological abnormalities, hydronephrosis, stones, masses, or recent use of nephrotoxic substances (five cases), and individuals with image artifacts or other factors that could potentially impact the texture analysis (30 cases).

**Figure 1:**
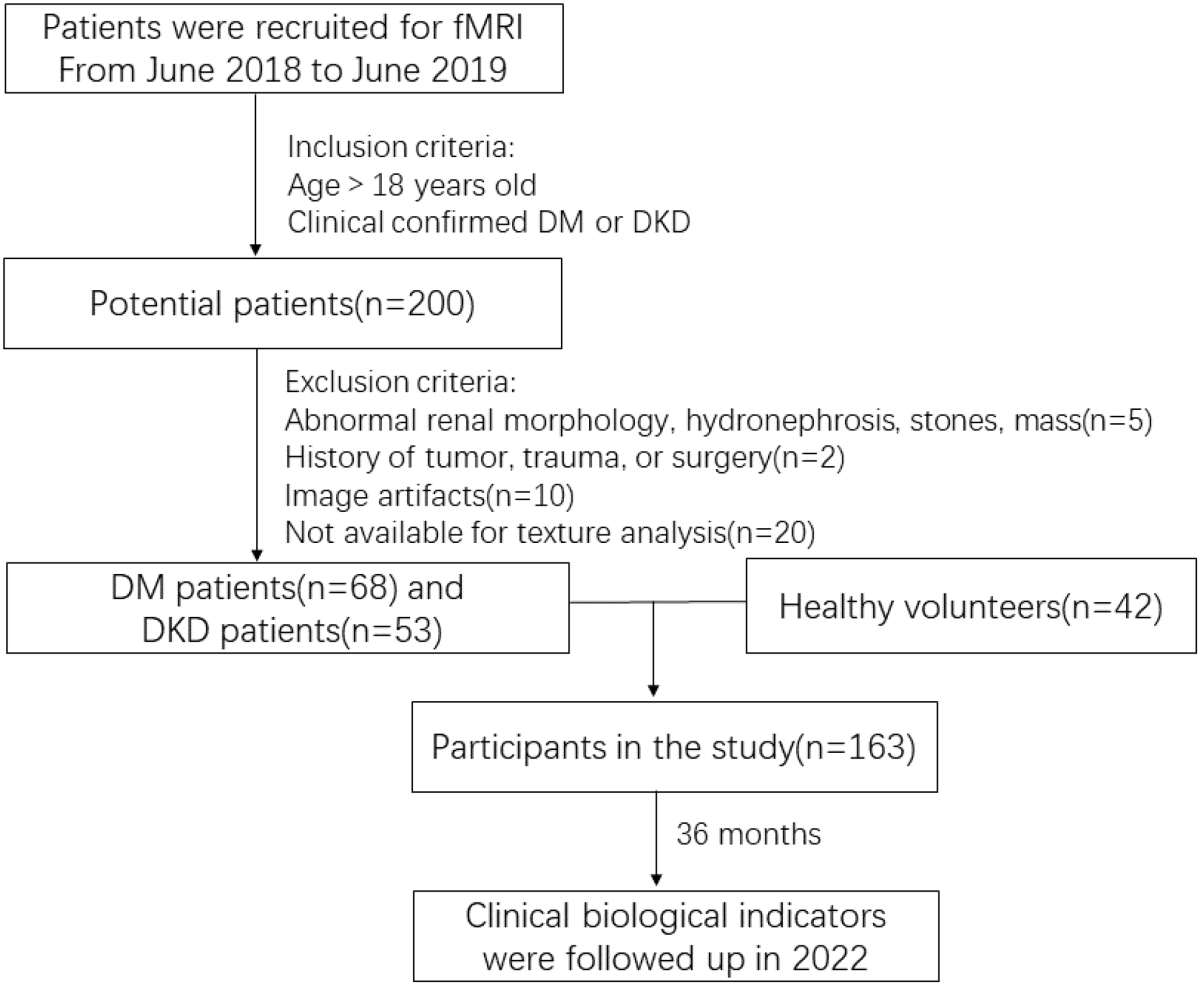
Study screening flowchart.

Ultimately, 163 individuals with an average age of 51 ± 12.21 years, consisting of 98 men and 65 women, were enrolled in the study. The screening process is depicted in **Figure 1**.

### Magnetic resonance examination

During functional MRI (fMRI) scanning, a 3.0T Philips Ingenia MRI scanner was utilized, with signal reception facilitated by 12 elements from the integrated spine matrix coil and six elements from the body matrix coil. The participants were positioned in a supine posture on the examination bed, instructed to breathe freely, and advised to minimize body movement during the scan. The scan was conducted by a proficient MR technician. The ASL scan parameters for the renal coronal sequence included a repetition time (TR) of 3200 ms and an echo time (TE) of 16 ms, AP = 10 mm, FH = 257 mm, RL = 316 mm for the field of view (FOV), 88×117 for the matrix size, and 10 mm for the slice thickness (THK) and gap of 1 mm. Post-processing of the original image data was conducted using a Philips workstation to generate the ASL image.

### Postprocessing, image segmentation, and extracting features

**Figure 2:**
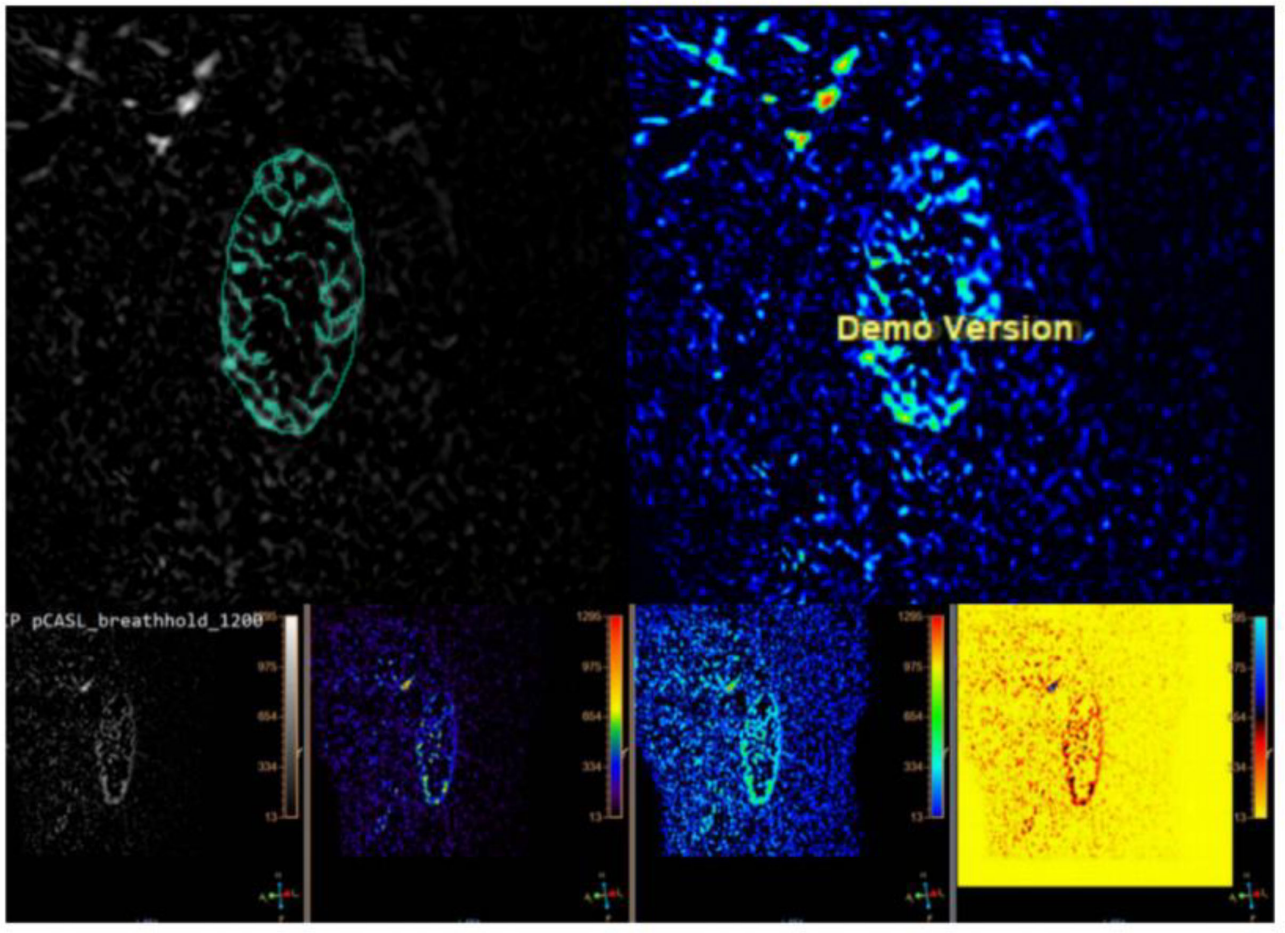
The ROI in the ASL image includes the renal cortex.

On the fMRI images, the radiomics features associated with diabetic kidney damage were analyzed, and the specific steps were as follows:

1. The program MaZda, which was created for the analysis of image textures, was used to load the initial ASL data (Version 4.6, https://www.eletel.p.lodz.pl/mazda) (15). Regions of interest (ROIs) were manually delineated to segment the ASL images. The radiologist manually outlined ROIs on ASL images of both the right and left kidney, encompassing the cortex (**Figure 2**).
2. To mitigate the impact of variations in contrast and brightness on analysis outcomes, prior to extracting texture parameters in MaZda, gray levels were normalized within the range [μ - 3δ, μ + 3δ], encompassing image intensity normalization and gray level discretization.
3. The MaZda software extracted texture features from the ROI, encompassing a total of 367 parameters, consisting of the run-length matrix (RLM), absolute gradient (GRA), co-occurrence matrix (COM), autoregression model, and Haar wavelet in addition to the gray-level histogram. The MaZda software used techniques such such as the Fisher coefficient, mutual information (MI), probability of classification error, and average correlation coefficient (POE + ACC), mutual information (MI), and classification error probability to determine the texture parameters.

### Data processing

Perfusion-weighted pictures were obtained by subtracting the control from the label images after motion correction. The equation below, which was developed from a single-compartment ASL perfusion model, was used to quantify renal blood flow from this map in milliliters per hundred grams per minute (16).


$$RBF=\frac{\text{Δ}M}{M_{0}}\frac{\lambda }{2\alpha }\frac{R_{1\alpha }}{\exp\left(-\omega R_{1\alpha }\right)-\text{exp}\left(-(\tau +\omega )R_{1\alpha }\right)}$$

The blood/tissue water partition coefficient was 0.9 mL/g; the labeling efficiency was 74%; the labeling duration was 1.6 s; the postlabeling delay was 1.2 s; the longitudinal relaxation rate of arterial blood, R1a, was 0.67 s^-1^; and the image intensity of the mean control image is Mo. The signal difference between the label and control images is represented by M.

### Statistical analysis

The clinical biological indicators in the three groups were statistically compared using multivariate analysis of variance (MANOVA) for the normally distributed data and the Kruskal-Wallis test for the non-normally distributed data. A significance threshold of p<0.05 was utilized. Image rendering was conducted with GraphPad Prism 8 and R Studio.

LASSO was employed to select the most informative texture characteristics. A refined model was achieved through the construction of a penalty function, which compressed the regression coefficient to zero and optimized the model parameters via cross-validation. The model was trained using a 10-fold cross-validation approach to predict early identification of diabetic kidney injury in patient groups A, B, and C. Seven machine learning algorithms were utilized to establish prediction models, with the optimal model for predicting various stages of kidney damage in individuals with DM selected.

The study utilized hierarchical clustering heat maps generated using R language and Python to visually represent the most valuable texture features and clinical biological indicators selected. Spearman correlation analysis was conducted to assess the relationship between the texture features and clinical indicators. The predictive capability of the model for different stages of diabetic kidney impairment was evaluated through ROC curve analysis using Python and MedCalc 15.8.

## Results

### Clinical characteristics of the study subjects

A total of 163 individuals underwent renal ASL, with 53 in group A, 68 in group B, and 42 in group C. Demographic and clinical variables are reported in **Table 1**. The resulting images met the diagnostic criteria. There were no statistically significant differences in gender or age among groups A, B, and C (p > 0.05).

**Table 1:** Baseline characteristics of different diabetic subjects.

	HVs(n=42)	DM(n=68)	DN(n=53)	*F*/*H* value	*P* value
Female n(%)	18 (42.8%)	29 (42.6%)	18 (34%)	7.130	1.458
Age (years, $\tilde{X}$ ± SD)	46.381 ± 12.165	50.941 ± 12.456	55.566 ± 10.613	1.140	0.001
BMI(kg/m^2^, $\tilde{X}$ ± SD )	23.671 ± 3.039	28.494 ± 3.622	26.852 ± 3.142	27.333	<0.01
SBP (mmHg, $\tilde{X}$ ± SD)	108.5 ± 7.178	133.632 ± 11.398	134.679 ± 18.883	55.729	<0.01
DBP (mmHg, $\tilde{X}$ ± SD)	74.167 ± 7.708	81.088 ± 8.954	81.981 ± 10.148	10.278	<0.01
ALT [U/L,M(Q1,Q3)]	30.45(22.475,40.025)	24.25(18.825,44.75)	20.5(14.5,31.8)	9.408	0.009
AST [U/L,M(Q1,Q3)]	27.25(21.175,34.75)	21.65(16.925,28.8)	21(15.85,27.9)	9.542	0.008
ALB(g/L)	48.410 ± 4.478	44.201 ± 4.618	41.691 ± 5.087	23.717	<0.01
AG	1.510 ± 0.157	1.645 ± 0.264	1.468 ± 0.247	9.379	<0.01
BUN [mmol/L,M(Q1,Q3)]	5.65(4.675,7.05)	5.08(4.26,6.263)	6.87(5.55,9.29)	27.350	<0.01
SCr [umol/L,M(Q1,Q3)]	61.65(56.15,65.925)	63.3(54.575,72.15)	74.7(63.45,123.25)	43.239	<0.01
UA [umol/L, M(Q1,Q3)]	271.2(247.225,293.575)	335.25(260.6,389.25)	382.2(315.8,427.65)	40.422	0.043
eGFR [ml/min/1.73m^2^, M(Q1,Q3)]	107.78(100.915,116.6775)	105.978(99.4,112.99)	84.51(50.635,102.33)	43.239	<0.01
UMA [mg/24h,M(Q1,Q3)]	9.75(3.1,14.45)	11.88(6.7,29.2)	110.1(56.015,1082.665)	85.683	<0.01
Pro	0(0,0)	0(0,0)	1(0,2)	70.778	<0.01
ACR [mg/g, M(Q1,Q3)]	6.9(5.5,8.725)	9.333(6.275,13.374)	223.21(43.505,223.21)	92.915	<0.01

### RBF values analysis of ASL imaging

**Figure 3:**
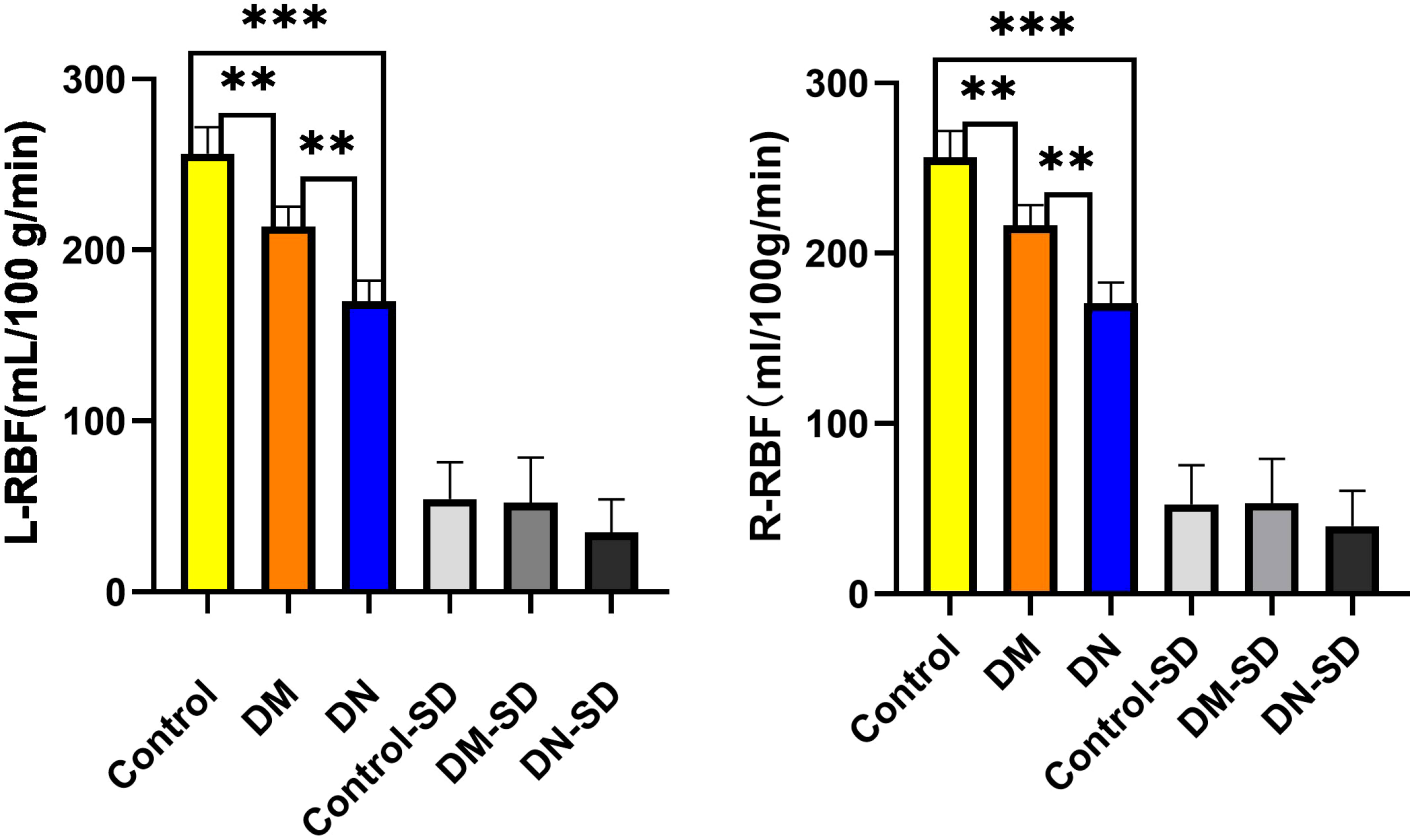
RBF values from ASL imaging in groups A, B and C. (*p<0.05,**p<0.01, ***p<0.001).

All participants underwent abdominal functional magnetic resonance imaging, and renal cortical blood perfusion values were assessed using ASL imaging. The bilateral RBF values in groups A, B, and C exhibited a progressive decrease, with values of 256.458 ± 54.256mL/100g/min, 213.846 ± 52.109mL/100g/min, and 170.204 ± 34.992mL/100g/min, respectively, as shown in **Figure 3**.

Statistically significant differences (p< 0.05) were observed in the RBF values among the various groups and within the three distinct groups. No significant disparity was noted in the RBF levels of the left and right kidneys. Furthermore, a positive correlation was identified between the RBF values and eGFR (r = 0.439, P< 0.001), while negative correlations were found with urinary albumin-to-creatinine ratio (UACR) (r = -0.691, p< 0.001), age (r = -0.242, p< 0.001), body mass index (BMI) (r = -0.253, p< 0.001), diastolic blood pressure (DBP) (r = -0.307, p< 0.001), blood urea nitrogen (BUN) (r = -0.245, p< 0.001), and serum creatinine (SCr) (r = -0.338, p< 0.001).

### Feature selection and extraction and the creation of a radiomics diagnostic model and radiomics signature

**Figure 4:**
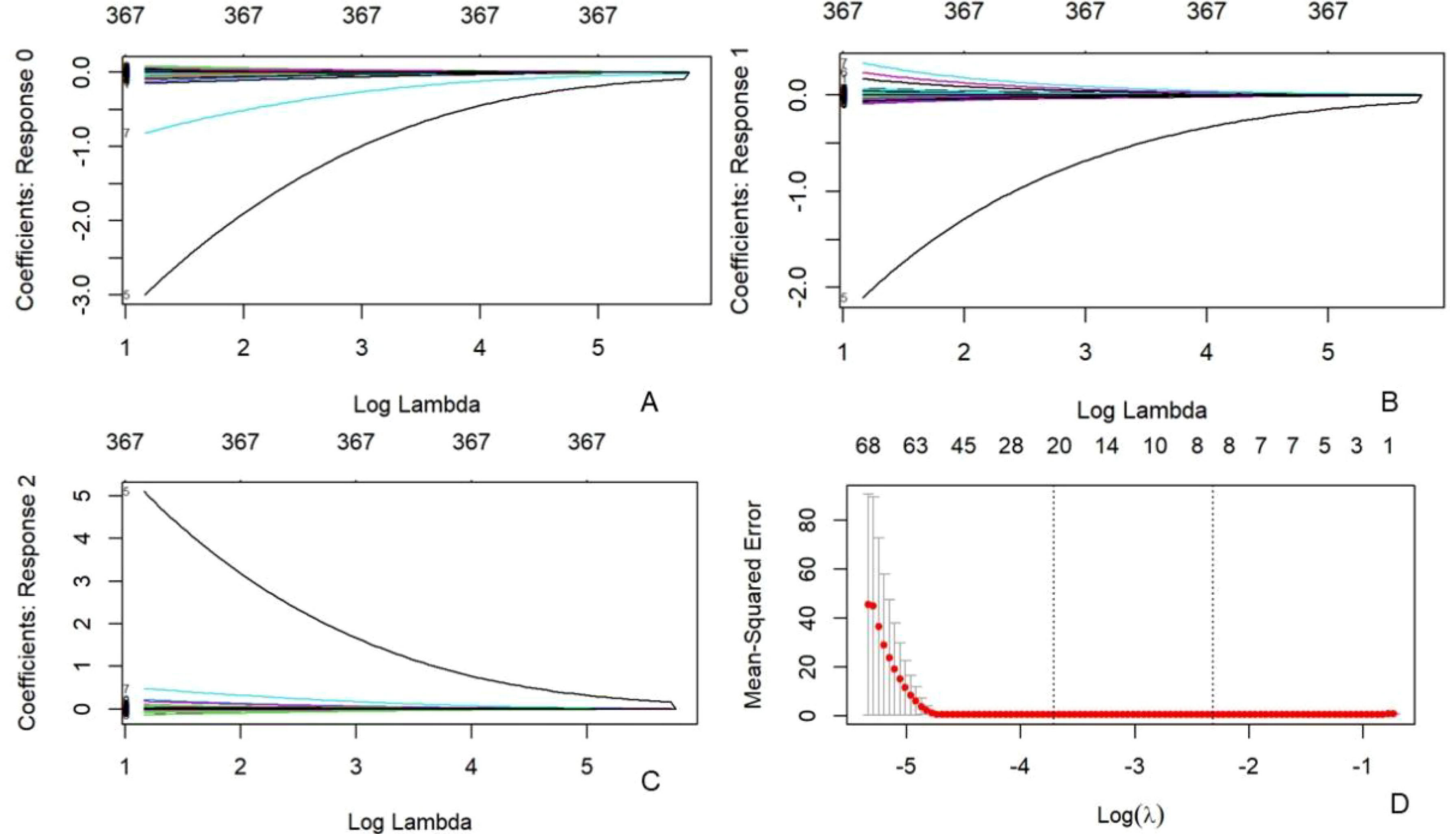
Texture feature selection was conducted using the LASSO binary regression model. The LASSO model uses 10-fold cross-validation to select the tuning parameter (lambda) and plot the binomial deviation curve against log(lambda). Vertical lines are drawn at the ideal values using the minimal criteria and one standard error method. This approach was used to screen eight texture characteristics. Texture features logit(P) = logit(P) = - 0.005483×GeoY + 0.215022×GeoRf - 0.099669×GeoRff + 2.829132×GeoRh + 0.657665×GeoW8 + 0.315119×GeoW12- 0.254502×S(0,4)Entropy - 0.073115×S(5,-5)Entropy.

Utilizing the MaZda software, a total of 367 texture characteristics were extracted from each kidney. Three algorithms, namely the Fisher Coefficient, Mutual Information, and Probability of Error plus Accuracy were utilized to identify the texture parameters of significance for further analysis. Subsequently, the eight texture features with the highest diagnostic values were selected for further investigation, as shown in **Figure 4** and **Table 2**. A LASSO regression model was then developed using the “glmnet” and “e1071” packages.

**Table 2:** Eight significant textural characteristics identified by the LASSO regression model.

Texture feature	Group A	Group B	Group C	*F* value	*P* value
GeoY	163.172(81.298~219.885)	142.743(64.222~201.104)	103.971(57.6543~211.164)	64.88	0.001
GeoRf	0.530(0.336~1.547)	0.670(0.373~1.529)	0.923(0.420~2.571)	54.73	0.001
GeoRff	0.456(0.270~0.684)	0.541(0.363~0.927)	0.677 (0.302~0.923)	74.29	0.001
GeoRh	0.934(0.872~0.983	0.953(0.899~0.983)	0.955(0.906~0.991)	29.81	0.001
GeoW8	0.462(0.270~0.702)	0.561(0.363~1)	0.734(0.308~1)	75.46	0.001
GeoW12	0.577(0.072~1.923)	1.074(0.147~1.838)	1.411(0.159~2.522)	66.36	0.009
S(0,4) Entropy	2.397(1.074~2.897)	2.16(1.157~3.048)	2.042(0.173~2.710)	22.94	0.007
S(5,-5)Entropy	2.291(1.415~2.877)	2.093(1.180~2.997)	1.976(0.184~2.666)	18.27	0.001

MANOVA and a nonparametric test were used to compare the texture features of the three groups. A statistically significant value was p < 0.05.

### Evaluation of the detection model

Based on the prominent indices of LASSO regression, eight optimal texture features were selected, and distinct machine learning algorithms were utilized to establish radiomics texture feature prediction models for renal damage in diabetic patients at various stages. The categorical variables were defined as follows.The positive variable was the patients with diabetes in Group B, and the negative variable was the healthy controls in Group A. At the same time, the positive variable was defined as diabetic nephropathy patients in Group C, and the negative variable was defined as patients with diabetes in Group B.

**Figure 5:**
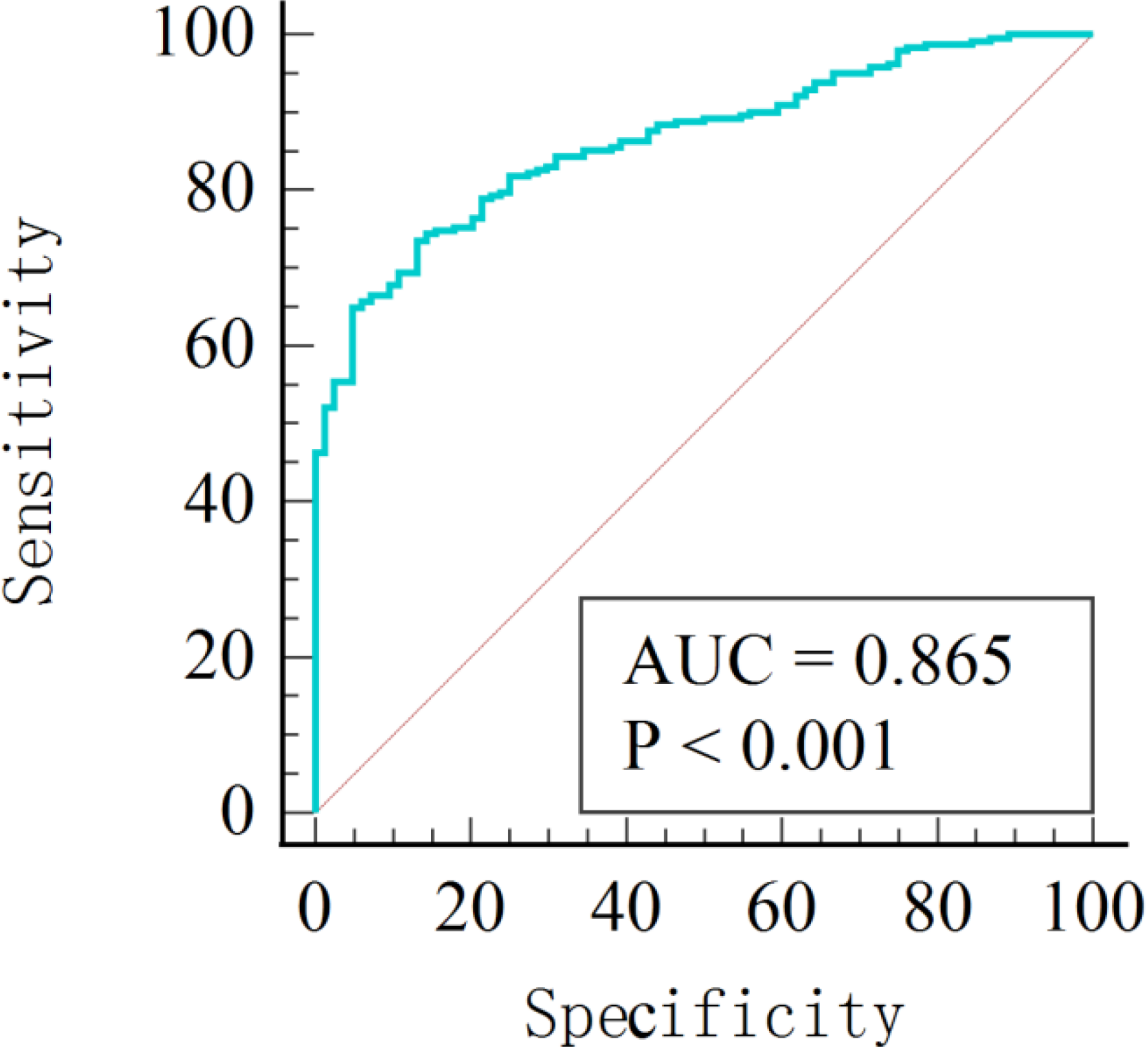
The diagnostic model’s degree of discrimination was evaluated using the AUC. This provided a paradigm for diagnosing different stages of diabetic kidney disease.

The area under the receiver operating characteristic curve (AUC) was computed for each participant to assess the discriminatory performance of the model. The most significant texture features in the model had an AUC of 0.865, a sensitivity of 85.1, and a specificity of 65.5 (**Figure 5**).

### Model development

**Figure 6:**
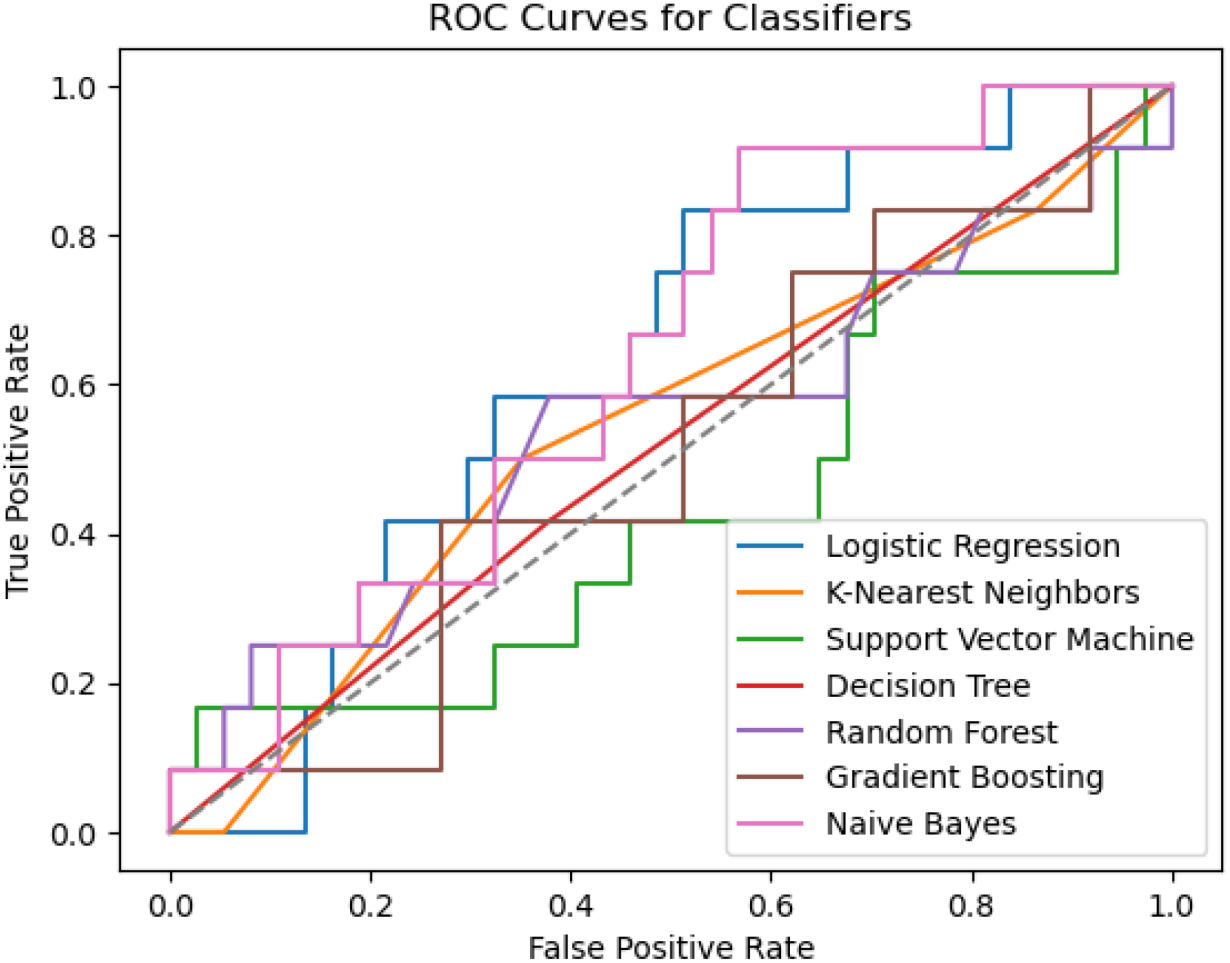
Prediction results of the machine learning algorithms.

Seven machine learning techniques, including decision trees (DTC), gradient boosting machines (GBM), logistic regression (LR), random forest (RF), K-Nearest Neighbor (KNN), support vector machines (SVM), and naive Bayes (NB), were employed to identify key characteristics and develop a radiomics texture feature prediction model. The subjects were divided into training (75%) and validation (25%) groups at random. Thus, 75% of the data were utilized for model construction, with the remaining 25% allocated for internal validation. A 10-fold cross-validation was conducted on the Training Set, representing 75% of the entire dataset. In total, 90% of the Training Set was utilized for training, while the remaining 10% was reserved for testing purposes. Among the seven classifiers examined, the NB classifier demonstrated the most favorable performance in the prediction model, with an accuracy of 0.74 and a precision of 0.43. The accuracy of the seven machine learning methods is depicted in **Figure 6**.

### Correlation analysis between texture features and clinical biological indicators

**Figure 7:**
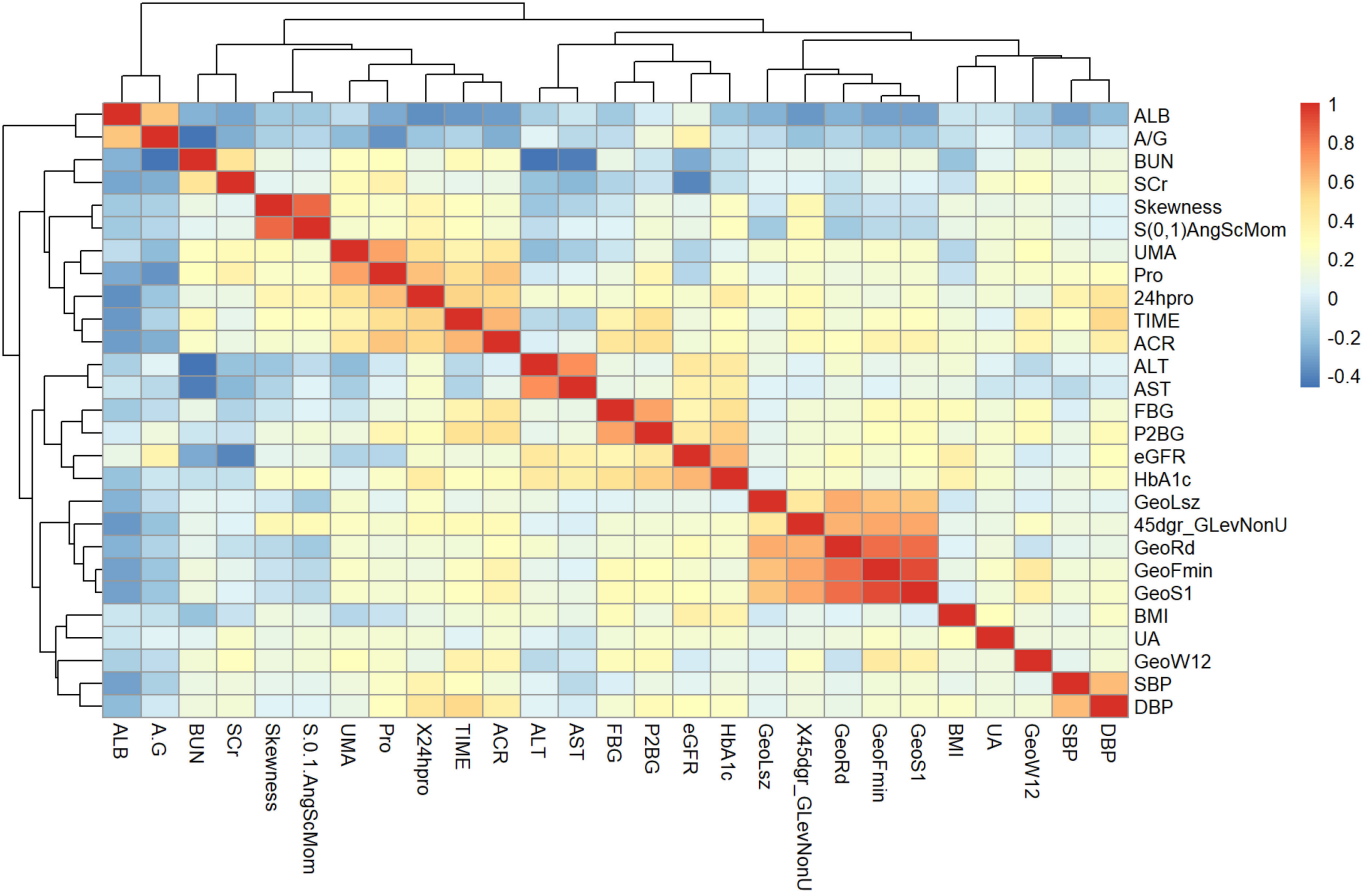
Heat map with meaningful hierarchical clustering of texture features and clinical biological indicators.

In order to examine the correlation between the eight pertinent textural characteristics and clinical biological markers, Spearman correlation analysis was employed. The data exhibited a skewness distribution in the normal distribution diagram and the Shapiro-Wilk test, while the scatter diagram (**Figure 7**) displayed a monotonic relationship between the two variables.

The identified texture feature parameters demonstrated varying degrees of significant correlation with eGFR and blood pressure measurements, including DBP, systolic blood pressure (SBP), albumin (ALB), albumin-to-creatinine ratio (ACR), BMI, fasting blood glucose (FBG), two hours postprandial blood glucose (P2BG), 24-hour urine protein, blood urea nitrogen (BUN), SCr, uric acid (UA), and microalbuminuria (UMA) in individuals with early diabetic kidney impairment.

## Discussion

Prompt detection and intervention in diabetic nephropathy hold considerable clinical significance. Previous studies have demonstrated a relationship between RBF, as evaluated by ASL, and the severity of diabetic kidney damage. In our study, we examined the textural characteristics of renal ASL images in a cohort of healthy individuals and those with varying stages of DM. Our findings suggest that radiomics features derived from ASL imaging can effectively identify both early and advanced stages of renal injury in patients with DM. To the best of our knowledge, our findings represent an initial demonstration of the efficacy of radiomics-based analysis in the quantitative evaluation of diabetic kidney ASL MRI. Furthermore, we examined the correlation between radiomics and clinical biological indicators.

DKD exhibits heterogeneity and lacks a consistently predictable clinical progression. eGFR and albuminuria are the two recognized biomarkers currently used to determine the stage of DKD. Existing literature has extensively discussed the limitations of these biomarkers (17–21). Moreover, formulating a personalized therapeutic approach solely based on these two biomarkers has proven challenging (22).

Previous studies have consistently demonstrated a positive association between ASL and eGFR in native kidneys (23–25). The utilization of radiomics in renal ASL has not yet been documented in the literature. Nevertheless, the analysis of texture feature parameters can enhance the assessment of kidney damage in patients with DM at various stages, leading to improved treatment strategies and efficacy evaluation.

A total of 367 texture feature parameters were extracted from ASL images of the left and right kidneys using MaZda 4.6 software. The selection of the most valuable texture parameters was conducted through LASSO regression analysis, following the refinement of 30 optimal texture feature parameters using the B11 statistical module within the MaZda program. Subsequently, models were developed for the evaluation of kidney damage in individuals with different stages of DM employing a range of machine learning techniques.

Tibshirani (26) was the first to propose the LASSO method. The evaluation of various machine learning algorithms, such as LR, RF, KNN, SVM, GBM, NB, and DTC, were performed in this study. After conducting a comparative analysis of the effectiveness of these machine learning algorithms in developing radiomics texture feature models for individuals with both DM and DKD at various stages, the results indicate that several classifiers utilizing the chosen features demonstrated optimal performance. The LR and NB models exhibited the highest AUC, sensitivity, positive predictive values, and negative predictive values. The LR algorithm identified eight primary factors for the risk prediction model: GeoFmin, GeoS1, GeoLsz, GeoRd, GeoW12, Skewness, and S(0,1)AngScMom,45dgr_GLevNonU. In the present study, the incorporation of the radiomics texture features alongside the clinical variables yielded a marginal improvement in the AUC-ROC. Despite the observed correlations among these features, the progressive addition of radiomics features bolstered the predictive capacity of each model for DKD and its rapid progression. As a result, the LR detection radiomics model and the NB prediction model were ultimately chosen for further analysis (p< 0.05), indicating a statistically significant disparity between the anticipated and observed outcomes. In order to evaluate the precision of the model, a comparison was made between the projected value and the actual value. The model was utilized to identify kidney damage in patients across three clinical trial datasets: a healthy control group, an early kidney injury group, and a diabetic nephropathy group.

The observed changes in texture features are suggestive of renal injury in diabetic patients at different stages indicates discrepancies in the structure and function of diabetic kidneys. This phenomenon may be attributed to the atrophy of renal tubules and the diminished capillary count in diabetic individuals, leading to a reduction in blood flow within the renal cortex. Wang et al. (27) indicated an association between kidney cortical perfusion and peritubular capillary densities, with interstitial fibrosis being identified as a factor that decreases cortical perfusion by disrupting peritubular capillaries.

Various confounding factors that impact cortical perfusion as measured by ASL should be taken into account. One such factor is anemia, which can potentially decrease brain perfusion measurements obtained through ASL due to the technique’s reliance on circulating blood as an endogenous tracer. This phenomenon may help elucidate the lower ASL values observed in the unstable graft group. It is worth noting, however, that anemia is commonly present in individuals with compromised kidney function. Additionally, variables such as the use of anti-hypertensive medications may also influence renal perfusion (28). These criteria, however, had no impact on our findings because the groups were matched according to these factors.

ROC curve analysis confirmed the effectiveness of using ASL for detecting kidney injury in patients with DM over time. The high AUC and specificity support this approach. First, it integrated various texture feature parameters associated with diabetic nephropathy into an assessment model, enabling the estimation of alterations in RBF and microstructure across patients with DM in different stages. This introduces a novel approach for clinicians, suggesting the need to integrate imaging markers to guide intervention strategies. Second, discerning interdependencies among markers proves challenging for clinicians. This system may also be used as a customized patient monitoring tool, using machine learning models that adapt to changing measurements through retraining.

More importantly, the high specificity of this distinction can be achieved by using ASL radiomics parameters, and a combination of biological and radiomics markers may be more useful for the follow-up of renal function changes.

While our findings highlight the potential utility in distinguishing radiomics texture features between healthy kidneys and various stages of kidney damage, our study does possess certain limitations. This study lacks a multicenter design and further research is needed to evaluate the diagnostic effectiveness of the ASL map-based radiomics model. Despite the small sample size, statistical variations in texture feature values were significant. ASL with free-breath scanning may have slightly lower image quality compared to breath-triggered images, but it offers advantages such as easier patient cooperation and shorter scan times.

## Conclusion

It is widely recognized that obtaining pathological results for early DKD in a clinical setting poses challenges, and quantitatively monitoring changes in renal function and structure is currently not feasible. In conclusion, we demonstrated that ASL sequences have the ability to sensitively detect alterations in kidney function and blood flow in patients with DM. Significant associations were observed between ASL imaging RBF values and clinical biomarkers. RBF values have the potential to identify early microstructural changes in the kidneys of DM patients. A model that combines imaging texture feature parameters with clinical biological indicators can predict early renal damage and progression in patients with DM early and intelligently.

## Data Availability

The original contributions presented in the study are included in the article/supplementary material. Further inquiries can be directed to the corresponding authors.
